# The clinical utility of DNA-based screening for fetal aneuploidy by primary obstetrical care providers in the general pregnancy population

**DOI:** 10.1038/gim.2016.194

**Published:** 2017-01-12

**Authors:** Glenn E. Palomaki, Edward M. Kloza, Barbara M. O’Brien, Elizabeth E. Eklund, Geralyn M. Lambert-Messerlian

**Affiliations:** 1 Department of Pathology and Laboratory Medicine, Women & Infants Hospital, Providence, Rhode Island, USA; 2 Department of Pathology and Laboratory Medicine, Alpert School of Medicine at Brown University, Providence, Rhode Island, USA; 3 Department of Obstetrics and Gynecology, Women & Infants Hospital, Providence, Rhode Island, USA; 4 Current affiliation: Department of Obstetrics and Gynecology, Beth Israel Deaconess Medical Center, Boston, Massachusetts, USA

**Keywords:** cell free DNA, clinical utility, Down syndrome, patient education, prenatal screening

## Abstract

**Objective:**

To assess the clinical utility of cell-free DNA (cfDNA)-based screening for aneuploidies offered through primary obstetrical care providers to a general pregnancy population.

**Methods:**

Patient educational materials were developed and validated and providers were trained. Serum was collected for reflexive testing of cfDNA failures. Providers and patients were surveyed concerning knowledge, decision making, and satisfaction. Pregnancy outcome was determined by active or passive ascertainment.

**Results:**

Between September 2014 and July 2015, 72 providers screened 2,691 women. The five largest participating practices increased uptake by 8 to 40%. Among 2,681 reports, 16 women (0.6%) were screen-positive for trisomy 21, 18, or 13; all saw genetic professionals. Twelve were confirmed (positive predictive value (PPV), 75%; 95% CI, 48–93%) and four were false-positives (0.15%). Of 150 failures (5.6%), 79% had a negative serum or subsequent cfDNA test; no aneuploidies were identified. Of 100 women surveyed, 99 understood that testing was optional, 96 had their questions answered, and 95 received sufficient information. Pretest information was provided by the physician/certified nurse midwife (55) or office nurse/educator (40); none was provided by genetic professionals.

**Conclusion:**

This first clinical utility study of cfDNA screening found higher uptake rates, patient understanding of basic concepts, and easy incorporation into routine obstetrical practices. There were no reported cases of aneuploidy among cfDNA test failures.

*Genet Med* advance online publication 12 January 2017

## Introduction

Clinical validity and clinical utility were first applied to genetic testing by the US Department of Health and Human Services’ Secretary’s Advisory Committee on Genetic Testing.^1^ These concepts were further developed in projects such as the ACCE model^2,3^ (analytic validity, clinical validity, clinical utility, and ethical, legal, and social implications; **Supplementary Figure S1** online, **Supplementary Table S1** online) and the Evaluation of Genomic Applications in Practice and Prevention project sponsored by the Centers for Disease Control and Prevention.^4^ Studies documenting the clinical validity of screening tests focus on quantifying the detection and false-positive rates under controlled conditions (e.g., karyotype-confirmed outcomes, case/control, or high-risk setting). Often, these studies are performed in settings that do not represent clinical testing (e.g., bulk testing of stored samples, omission of patient reporting, little or no retesting of failures). However, studies of clinical utility are designed to be performed in a clinical care setting (e.g., patients informed of testing options, clinical test results returned and used in patient decision making). In addition to verifying test performance as determined by case/control or retrospective cohort studies, clinical utility studies can also examine process-related components of implementation such as provider education and experience, patient education and knowledge, screening uptake rates, and women’s decision making. They can also explore other issues such as the economics of screening, long-term program evaluation, and availability of suitable facilities.^3^

Integrated screening is the most effective serum-based test for Down syndrome (90% detection rate, 3% false-positive rate), with a positive predictive value (PPV) of 6% in the general pregnancy population.^5^ In 1997, cell-free DNA (cfDNA) was found in maternal circulation,^6^ and next-generation sequencing (NGS) enabled proof-of-concept studies identifying common fetal aneuploidies in 2008.^7,8^ In 2011, the first external clinical validation study reported 98.6% of 212 Down syndrome pregnancies were screen-positive, 0.2% of euploid pregnancies were false-positives, and 0.8% resulted in test failures (no calls) after duplicate sample testing.^9^ This test efficiency has been confirmed by others.^10^ The term “cfDNA screening” here refers to the NGS of placental and maternal DNA fragments in maternal plasma to identify common fetal aneuploidies (aka “noninvasive prenatal screening” (NIPS)^11,12^). After defining the term “cfDNA screening,” we used that term in all provider and patient communications, including presentations, educational materials, individual patient reports, and surveys.

In 2012, the American College of Obstetricians and Gynecologists (ACOG)^9^ recommended offering cfDNA as secondary screening in high-risk pregnancies, with diagnostic testing offered to those with a screen-positive or failed result. ACOG^13^ and others^14,15,16,17,18^ recommended against cfDNA screening in the “lower-risk” population pending more information. At that time, the American College of Medical Genetics and Genomics did not directly address testing based on risk stratification,^11^ although their most recent recommendations suggest offering testing regardless of initial risk.^12^ To avoid the imprecision regarding testing “low-risk” or “high-risk” populations, we examined the utility of cfDNA testing with primary screening in the general pregnancy population (including the 15 to 20% of women age 35 and older). No study has yet demonstrated that a complex molecular test such as cfDNA screening can be offered successfully through primary obstetrical care offices.

Our process-oriented project aimed to document several clinical utility aspects of cfDNA screening for common aneuploidies through the implementation of a statewide program called DNA*First*. DNA*First* would be offered through primary obstetrical care providers^19^ as a routine first-line prenatal screen for the general pregnancy population. The study’s funding source (Natera, San Carlos, CA) was not involved in study design, data collection or analysis, manuscript preparation, or final approval. There was no charge to patients or their insurance for the DNA*First* test (the cfDNA portion of testing was provided by Natera), ensuring that women’s decisions about choice of screening test (integrated versus DNA*First*) would not be influenced by patient out-of-pocket expenses. The observed false-positive rates, PPV, and failure rates could be compared with those derived from previous clinical validity studies. Clinical utility issues addressed included comparing screening uptake rates before and after introducing DNA*First*, evaluating an innovative reflexive serum testing protocol for cfDNA failures, and exploring women’s decision-making. A survey was included to document experience, knowledge, and choices made by a subset of enrolled women. Participating providers were also surveyed to assess their ability to include DNA*First* into routine practice and to identify perceived advantages and impediments.

## Materials and Methods

### Overview

The institutional review board at Women & Infants Hospital (WIH) approved the project (13-0013), which is registered with ClinicalTrials.gov (NCT01966991). The DNA*First* screen begins with cfDNA testing performed by a commercial laboratory using a SNP genotyping method (Natera)^20,21^ with reflexive serum/ultrasound screening in the event of cfDNA test failure. New DNA*First* patient materials specifically targeted for the general pregnancy population were developed, evaluated,^22^ and validated using an approach reported previously.^23^ Providers were offered a short in-service education program at each practice site. All pretest education was delivered to the pregnant women by primary obstetrical care providers in Rhode Island; logistics and materials resembled those of well-established serum screening protocols. Phlebotomists were trained and customized requisitions (**Supplementary Figure S3** online) and reports (**Supplementary Figure S4** online) were tailored for our local practices (e.g., all reports included a reminder that serum screening for open neural tube defects should be considered). The DNA*First* program focused on trisomies 21, 18, and 13, as well as monosomy X, because these are identifiable by current integrated screening. Interpreting cfDNA results for common sex trisomies (e.g., 47, XXY)^24,25,26^ is not recommended by ACOG^27^ but was included as a DNA*First* “opt-in” (including reporting the fetal sex). Women with screen-positive results were referred to the WIH Prenatal Diagnosis Center for genetic counseling and diagnostic testing. A subset of women with screen-negative or failed cfDNA tests was surveyed to learn about how DNA*First* test information was obtained, level of knowledge, satisfaction, and decision-making processes. Detailed methods are available in the supplement materials (**Supplementary Methods** online).

### Data collection and statistical methods

Active enrollment was designed to run for at least 9 months allowing time for providers to reach a “steady state” of screening. This was also considered sufficient time to accumulate a minimum of 10 autosomal trisomies. Follow-up test results (e.g., reflexive serum testing, cfDNA testing after failure on a subsequent plasma sample, diagnostic testing results, pregnancy outcomes, newborn karyotypes) were sought for women with screen-positive results or initial cfDNA test failures. The 95% confidence intervals (CI) of proportions were based on the binomial distribution (TrueEpistat, Round Rock, TX). Significance was two-tailed at the 0.05 level.

## Results

### Enrolling providers

**Figure 1:**
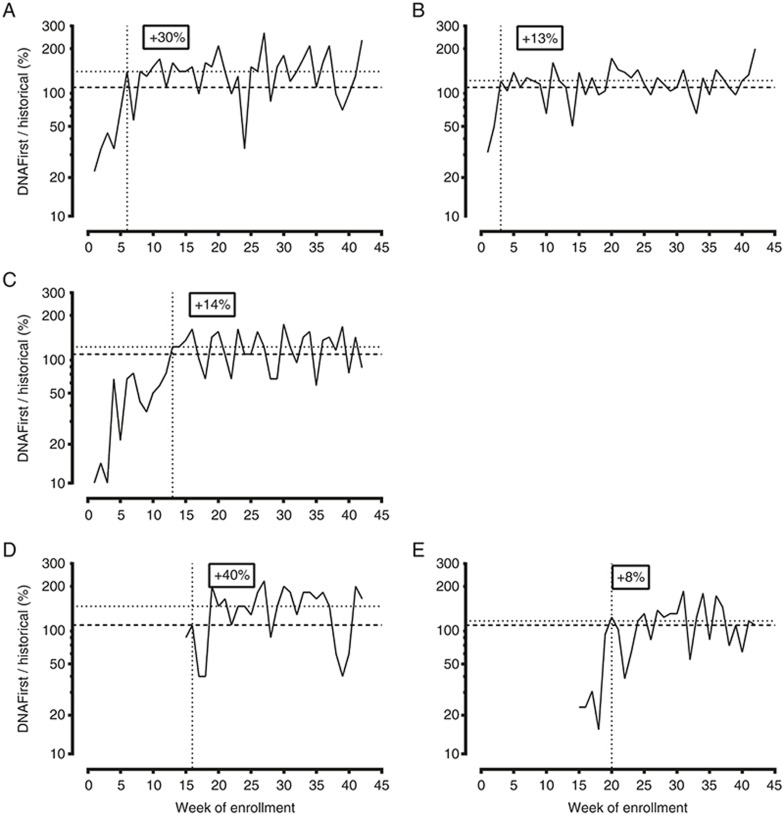
**Weekly DNA*First* test enrollment of the five largest participating practices, expressed as a percentage of historical serum screening.** Week of study enrollment (horizontal axis) versus weekly test volume (expressed as a percentage of serum screening volume in the previous 6 months). Practices A through C began enrolling soon after study initiation and exceeded historical screening rates by 30, 13, and 14% (horizontal dashed lines) by weeks 6, 3, and 13, respectively (vertical dashed lines). Practices D and E began enrollment later but matched historical rates quickly (at 16 and 20 weeks) and exceeded those rates by 40 and 8%, respectively.

Primary obstetrical care practices were approached in June 2014; five of the seven largest group practices in Rhode Island (>400 new patients per year) agreed to participate. Two declined, citing anticipated complexity and/or the 2012 ACOG recommendations against offering cfDNA screening to “low-risk” women.^13^ Subsequently, smaller practices were informed and encouraged to participate. Between September 2014 and July 2015 (11 months), 2,691 women agreed to undergo screening through 72 providers. The five large practices included 78% of all providers and accounted for 82% of the women screened. DNA*First* became their primary screen within 2 to 11 weeks after introduction (i.e., when weekly DNAFirst tests exceeded those for serum screening in the previous 6 months). All five large practices eventually exceeded historical serum screening rates by 8 to 40% (average, 18%) (**Figure 1**). Insufficient numbers of screened women in the smaller/solo practices precluded performing a similar analysis.

### Characteristics of screened women

**Figure 2:**
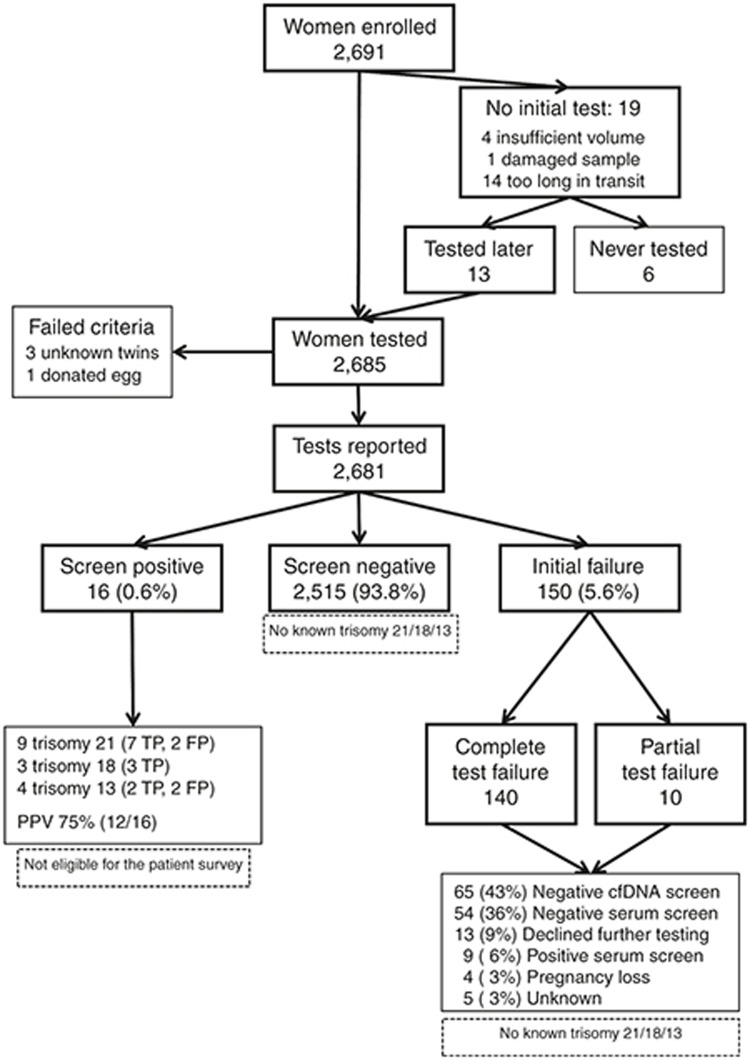
**Flowchart showing DNA*First* testing for trisomies 21, 18, and 13, along with additional testing for initial test failures and selected outcomes.** Overall, 2,691 women agreed to testing and 2,685 samples had DNA sequencing. Of the 2,681 cfDNA tests reported, 0.6% (16) were screen-positive, 5.6% (150) failed to report at least one chromosome, and the remaining 93.8% (2,515) were screen-negative. FP, false positive; TP, true positive.

**Figure 2** shows DNA*First* screening flow for the 2,691 women and focuses on trisomies 21, 18, and 13. Testing was not initiated for samples from 19 women, including 14 from a single lost shipment. Thirteen women submitted a second sample (68%); the remaining six did not. After cfDNA testing, four samples were ineligible due to unreported exclusion criteria (three dizygotic twins and one donated egg). Of the three twin pregnancies, one was known and submission of the sample was in error, another was unrecognized at the time, and details were unavailable for the third case. **Table 1** shows characteristics of the remaining 2,681 women. Median gestational age was 12 weeks, with 1.6% collected after 20 weeks. Of the 43 initial samples collected after 20 weeks, 29 (67%) were collected by 24 weeks, which was beyond our recommended limit of 20 weeks for the study but still considered acceptable clinical practice. None of the samples collected at 25 weeks or later had an abnormal ultrasound finding as an indication. Median maternal age was 31 years, with 21% age 35 years or older—a rate similar to the 17% who underwent serum screening in the previous 6 months. Self-reported race included 85% Caucasian, 6% African American, and 4% Asian American; 15% reported being of Hispanic ethnicity. Testing indication was primary screening for 88%, advanced maternal age for 10% (these were considered part of a general pregnancy population), and history of a spontaneous loss for 1%. Requisitions for only eight women (0.3%) reported abnormal ultrasound or abnormal serum screen results, supporting our contention that this cohort represents an unscreened general pregnancy population. We honored requests outside the recommended testing protocols if reliable testing was still possible (e.g., collection at 21 weeks was acceptable). This is in contrast to pregnancies with a donor egg, when testing using this methodology is not possible. No samples submitted for DNA*First* testing were excluded from this report.

### Screen-positive results for trisomies 21, 18, and 13

The cfDNA screen-positive rate for trisomies 21, 18, and 13 was 0.60% (**Figure 2**; 16/2,691; 95% CI, 0.34 to 0.97%). Of these, 11 were true positive and four were false positives; all were confirmed by invasive testing and diagnostic testing (e.g., karyotyping). The sixteenth result (screen-positive for trisomy 13) was clinically consistent with trisomy 13 (bilateral polydactyly, cystic hygroma, and spontaneous loss at 15 weeks with findings confirmed on abortus) but not karyotyped; it was also classified as a true positive. All 16 were referred to the WIH Prenatal Diagnosis Center and all were seen by genetic professionals. Nine true positives were prenatally confirmed and seven were terminated (78%). Based on maternal and gestational ages, 13.1 autosomal trisomies were expected^28^ (9.4, 2.8, and 0.9 for trisomies 21, 18, and 13, respectively) and 12 were identified (7, 3, and 2, respectively). The PPV was 75% (12/16; 95% CI, 48 to 93%) and the false-positive rate was 0.15% (4/2,681; 95% CI, 0.04 to 0.38%). Of the eight enrolled women with a previous abnormal ultrasound or serum screen result, one was screen-positive for monosomy X and confirmed.

### Screen-negative results

The screen-negative rate was 93.8% (**Figure 2**; 2,515/2,681); these were subject to passive ascertainment. Review of newborn and infant karyotypes at WIH identified no additional aneuploidies and none were reported from participating providers. However, we were made aware of two monozygotic twin pregnancies with screen-negative cfDNA tests (cfDNA testing using the SNP-based methodology does not identify monozygotic twins).

### Failed cfDNA testing

The initial cfDNA test failure rate was 5.6% (150/2,681; 95% CI, 4.8 to 6.5%) and all were subject to active outcome ascertainment. For the 85 plasma samples subsequently submitted for cfDNA testing, 65 (76%) results were reported; all were screen-negative (**Figure 2**). An additional 63 women relied on reflexive serum/ultrasound results; 54 (86%) were screen-negative and 9 (14%) were screen-positive. Eight of these nine women delivered a normal infant (four also had a subsequent screen-negative cfDNA test). The ninth woman was diagnosed with a mosaic condition after a positive cfDNA test from another sequencing laboratory; a normal female infant was delivered. **Figure 2** lists outcomes for the remaining pregnancies with test failures. Our follow-up revealed that none of these 150 women chose invasive testing. To verify provider awareness that open neural tube defect screening is indicated despite normal cfDNA test results, records from 100 women consecutively screened in May 2015 (near the end of the study) were reviewed: 72% had serum alpha-fetoprotein screening for open neural tube defects, with no screen-positive results (≥2.0 MoM). It was not possible to determine whether the 28% of women who did not undergo open neural tube defect screening were not offered serum screening, declined serum screening, or underwent alternative testing such as a level II ultrasound.

### Changes in testing over time

The numbers of providers and women screened increased over time, most rapidly in the first 6 months (**Table 2**). Three-quarters of samples shipped the day of collection, with a median turnaround of 10 days (sample collection to report received by provider); 95% of results were returned within 15 days. In the first 2 months, a higher failure rate was noted in 35% of samples collected at 10 weeks. The 60 failures due to low fetal fraction occurred more frequently at 10 weeks versus 11–21 weeks (risk ratio, 2.5; 95% CI, 1.3 to 4.5; P = 0.007). In month 3, this prompted a recommendation that the optimal earliest time for collection would be 11 weeks although 10-week samples would be accepted. Subsequently, less than 8% of samples were collected at 10 weeks (**Table 2**). DNA failures were also confirmed^29^ to be strongly associated with maternal weight of 80 kg or higher (risk ratio, 11.4; 95% CI, 6.3 to 21; P < 0.001; **Supplementary Figure S5** online). In months 7 and 8, 22 additional failures at 3 weeks were attributed to a laboratory reagent problem that raised the rate to 7.1%.

### Sex chromosome screening

All pregnancies were routinely screened for monosomy X and three (0.11%) were screen-positive (3/2,681; 95% CI, 0.03 to 0.33%). Two were true positives; both ended in spontaneous losses. The third resulted in a late-first-trimester fetal loss with no diagnostic information (**Supplementary Figure S6** online). Optional sex trisomy (and fetal sex) interpretations were chosen by 91.2% of the women (2,445/2,681). Two were screen-positive for a sex trisomy; both women received posttest genetic counseling and both declined prenatal diagnostic testing. Both infants were live-born; one was confirmed by postnatal karyotype. Thirteen additional sex chromosome failures occurred (0.5%). No discrepancies regarding the predicted fetal sex were reported.

### Surveys of screened women

The test requisitions of two-thirds of women (**Table 2**) included permission to be contacted (an institutional review board requirement); a pool of 140 was selected. Seven phone numbers were incorrect or out of service, and contact was unsuccessful for another 20. Of the remaining 113 women, 100 (88%) completed the 15-min survey after providing verbal consent. Interviews occurred 3 to 5 months after testing, but all women were still pregnant. This time frame was chosen to ensure that participants had completed all decision making about screening and follow-up prior to being contacted.

A complete list of responses to selected questions is shown in **Table 3**. Women reported receiving information from their physician or certified nurse midwife (55%) or an office nurse/educator (40%) in less than 5 min (36%) or in 5 to 9 min (39%). They reported sufficient time to talk with their provider (95%), having their questions answered (96%), and feeling that the optional nature of screening was conveyed (99%). Although 85% understood that the test identified Down syndrome, 15% thought it identified all genetic problems. Most (79%) understood that a negative result did not rule out Down syndrome but 13% thought it did. Overall, 69% knew that “the test could not tell for certain if the baby has Down syndrome”; however, 28% thought it could. Women were not nervous about testing (mean, 2.4; 1 = not at all, 5 = very) and 93% rated their decision as “good” or “great” (mean, 4.2; 1 = terrible, 5 = great). Nearly all (97%) remembered reviewing DNA*First* results with office personnel, 98% would recommend testing to friends, and 95% said they would undergo the test in their next pregnancy. They reported a willingness to pay $10 to $50 (38%) or $51 to $100 (33%) out of pocket. Eighty-seven women remembered making decisions regarding sex chromosome trisomy screening/fetal sex. The 78 women who chose such screening wanted to know the baby’s sex (77%), wanted as much information as possible (67%), and liked not being required to pay (47%). Of nine women who did not choose sex chromosome trisomy testing, eight did not want to know the fetal sex. Knowing fetal sex was “very important” for 46% and “not important for 34%”; 20% “did not want to know.” 

### Surveying the obstetrical care providers

Surveys were completed by 33 of 72 providers (46%) and included 21 physicians and 8 certified nurse midwives. Among physicians, 90% reported personally discussing DNA*First* with women. An average of 6 min was spent informing and answering women’s questions (range, 2.5–15 min), which was consistent with women’s estimates. Providers felt their staff was adequately prepared (83%) and that 60 to 100% of women they talked to about DNA*First* accepted screening. Respondents thought women accepted screening to reveal fetal sex (90%), receive better/more accurate results (28%), receive earlier results (14%), simplify screening (10%), and undergo testing at no charge (7%). Providers were positive about the ease of offering DNA*First*, screening program support, and test performance; however, they expressed concerns about the DNA failure rate, turnaround time, and costs of testing when the project ended.

## Discussion

This is the first report documenting multiple clinical utility aspects of a cfDNA-based prenatal screening test for common aneuploidies in a general US pregnancy population, offered through nonacademic, community-based obstetrical care practices. Patient educational materials were designed and validated specifically for use by the general population. The DNA*First* test (cfDNA coupled with reflexive serum screening) was designed to address test failures in a population at general risk and to examine patient interest in sex chromosome screening as a test option. The associated programmatic activities were coordinated through an experienced prenatal screening program whose structure was based on ACOG recommendations promulgated in 1982 that recommended a “coordinated system of care resulting in prompt, accurate diagnoses and appropriate follow-up services.”^30^

Concerns regarding the use of cfDNA in the general pregnancy population include the reliability of PPV estimates. Among our 16 trisomy 21, 18, or 13 screen-positives, the PPV was 75% (three true positives for each false positive or 3:1). These odds are 50 times higher than the 6% (1:17) achievable by integrated screening but 33 times lower than the >99% (>98:1) reported by several commercial laboratories. Individual risks or PPV of >99% are almost certainly overestimates because they do not account for rare clinical false-positive results that may even be analytically correct (e.g., confined placental mosaicism, vanished affected twin, maternal mosaicism, maternal cancer). Such high risks also tend to undermine the “screening” nature of this testing. Our PPV is consistent with the estimates reported from controlled clinical validity studies in the general pregnancy population.^31,32^

When screening in the general population, DNA test failures are a major concern. In the high-risk setting, women with test failures can be offered diagnostic testing due to their existing risk. It seems inappropriate to offer diagnostic testing to all women with a test failure in the general pregnancy population. For example, the risk of aneuploidy is likely to be quite low in a 21-year-old woman weighing 250 pounds whose test result is a failure due to low fetal fraction. We report a failure rate of 5.6%, which is at the lower end of the published rates for this methodology^6^ but is still high. For DNA*First*, a new blood draw was required for a repeat cfDNA analysis in the event of a test failure and, although it delayed final reporting, no aneuploidies were identified. Our innovative reflexive serum testing protocol worked as intended to provide an acceptable alternative to repeat testing.

Recently, both the ACOG^33^ and ACMG^12^ recommended that genetic counseling and comprehensive ultrasound and diagnostic testing be offered after an initial cfDNA test failure for both high-risk and general pregnancy populations.^33^ These recommendations were based on only three published studies.^21,32,34^ Of these, two did not perform routine repeat testing for all failures,^21,32^ but the other did.^34^ However, this latter study did not provide pregnancy outcomes among those failures. It is critical to distinguish between cfDNA test failure rates (and associated risk of aneuploidy) when only an initial test is performed versus those same rates after a duplicate or subsequent sample has been tested. Further analyses of the usefulness of repeat testing based on all relevant published studies are warranted.

Obstetrical care providers received in-person training and had program-specific, validated, and grade-appropriate patient educational materials available. Given this, our patient survey results indicated that most women understood the basic concepts of cfDNA screening. The patient and provider survey results were unique in that they focused on pregnant women from the general population choosing cfDNA testing as a clinical test after being informed by primary obstetrical care providers during routine clinical practice. None of the women received pretest education from genetic professionals because the lack of resources made this impractical. Such a practice would also deviate from established prenatal serum screening protocols. Although not perfect, levels of knowledge were at least as good as in studies of women undergoing genetic counseling for cfDNA screening^35,36,37^ and in older studies of women’s knowledge regarding serum screening.^38,39^

A recent study performed in Indiana^40^ reported a similar patient survey. In that study, 98 women with a screen-negative cfDNA test completed a questionnaire about their understanding. Nearly half (49%) said they “agreed” or “strongly agreed” with the (false) statement that “There is no longer a chance for my baby to have Down syndrome.” This contrasts with our survey, in which a similar question resulted in only 13% incorrect responses that there is “no chance” (another 3% reported “a 50/50 chance” and 5% said “didn’t know”). Our results are even more impressive given that most women in the Indiana population had high-risk pregnancies (67% were ≥35 years old, 20% had an abnormal ultrasound result) and many had formal genetic counseling.

Our study has limitations. The size of the group tested (2,681 women) allowed a confident estimate of only the false-positive rate (upper CI, 0.38%) and combined PPV (lower CI, 48% or >1:1). Also, the fact that there was no financial cost to the patient or her insurance may have resulted in higher uptake. However, our project was designed to simulate the low financial barriers to serum screening due to broad insurance coverage in Rhode Island. Such coverage may exist for cfDNA screening in the near future. We documented an average 18% higher uptake of DNA*First* than for serum screening among five large practices; a recent survey-based study found similar results.^41^ Unfortunately, we could not determine the reason for this. It may be related to the higher detection and lower false-positive rates, the ability to learn the fetal sex earlier in pregnancy, the availability of testing at no charge, the simplicity of offering one test over a wide gestational age range, or a combination of these or other factors. Regardless, the findings have implications for future economic analyses. cfDNA testing may have a higher uptake than current serum screening when offered to a general pregnancy population, leading to a higher proportion of cases detected in the population. We did not have access to measures of socioeconomic status, but all the enrolled group practices accepted Medicaid recipients. In our project, 13.1 common trisomies were predicted, 12 were identified, and none were found among the 150 initial test failures. There were four spontaneous losses among these 150 women and the occurrence of an unidentified trisomic loss cannot be ruled out. Our population was 85% Caucasian; this was the most common race indicated by the 15% self-reporting Hispanic ethnicity. Thus, the transferability to racial/ethnic groups such as blacks and Asians may be more limited.

This study contributes new information about the clinical utility of cfDNA sequencing of maternal plasma to screen for aneuploidy in the general pregnancy population (as described by the ACCE model; **Supplementary Figure S1** online, **Supplementary Table S1** online). We successfully implemented such screening with validated pretest educational information delivered by primary obstetrical care providers. The women were adequately informed and providers were able to integrate cfDNA screening into daily routines. The false-positive rate was confirmed to be very low and the PPV was confirmed to be much higher than that with current technologies. Test failures were adequately addressed through a combination of repeat cfDNA sampling and reflexive serum screening, and screening for neural tube defects continued successfully. We found higher failure rates at 10 weeks; this may suggest that an optimal window for general population screening is between 11 and 18 weeks of gestation, with samples at 10 or 19 weeks or later still being acceptable. Given that such a program has now been shown to be feasible, laboratories must strive to offer affordable cfDNA sequencing that third-party payers could routinely cover in order to improve access to better aneuploidy screening for the more than 2 million pregnant women in the United States currently choosing prenatal screening for Down syndrome.^42^

## Disclosure

The cell-free (cf) DNA tests were performed at no charge by a CLIA-approved and CAP-accredited commercial laboratory (Natera, San Carlos, CA). A sponsored research contract between Natera and Women & Infants Hospital provided partial support for study personnel and their activities. By contract, the funding source was not involved in study design, data collection or analysis, manuscript preparation, or manuscript approval. The authors and Women & Infants Hospital previously received grant funding from Sequenom (San Diego, CA) between 2008 and 2011 to perform an external validation study of their cfDNA test for common aneuploidies. G.E.P. is a statistical consultant to Beckman Coulter (Chaska, MN) and Ansh Laboratories (Webster, TX). G.E.P. and G.L.M. have performed research projects involving serum markers for PerkinElmer (Lexington, MA). All consulting and research were performed through contracts with Women & Infants Hospital. The other authors declare no conflict of interest.

## Figures and Tables

**Table 1:**
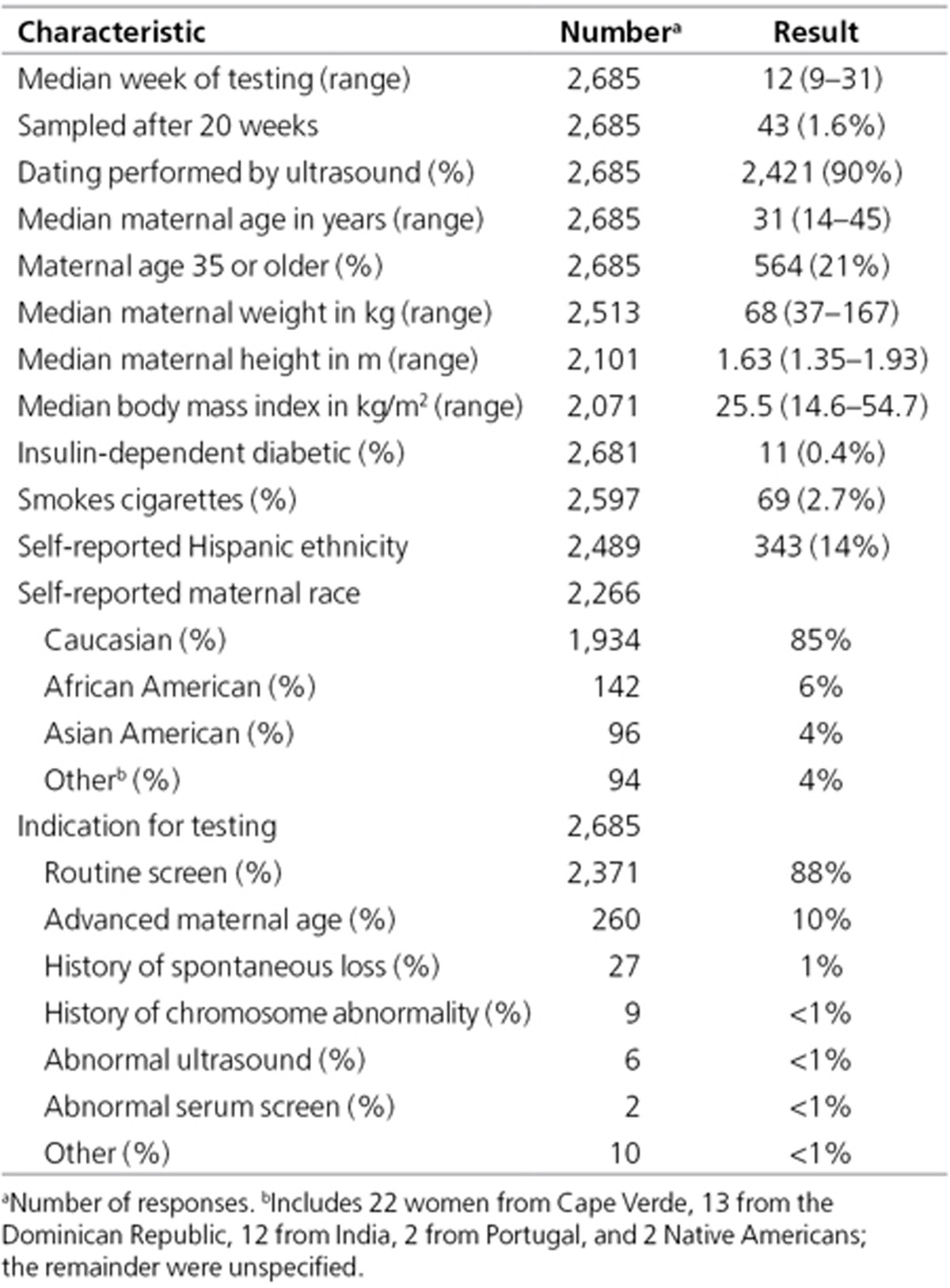
Characteristics of the 2,685 women who underwent DNA*First* testing in Rhode Island

**Table 2:**
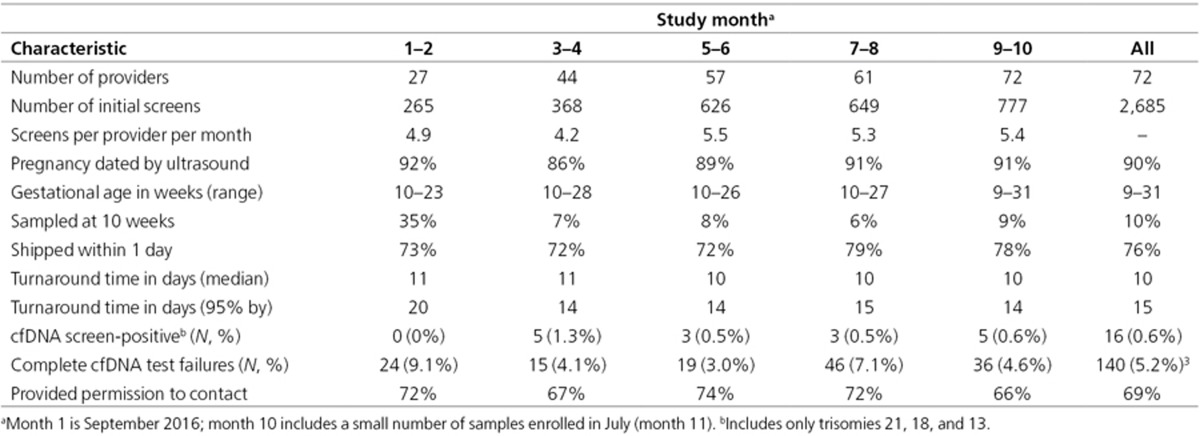
Changes over time in DNA*First* test characteristics and practice patterns

**Table 3:**
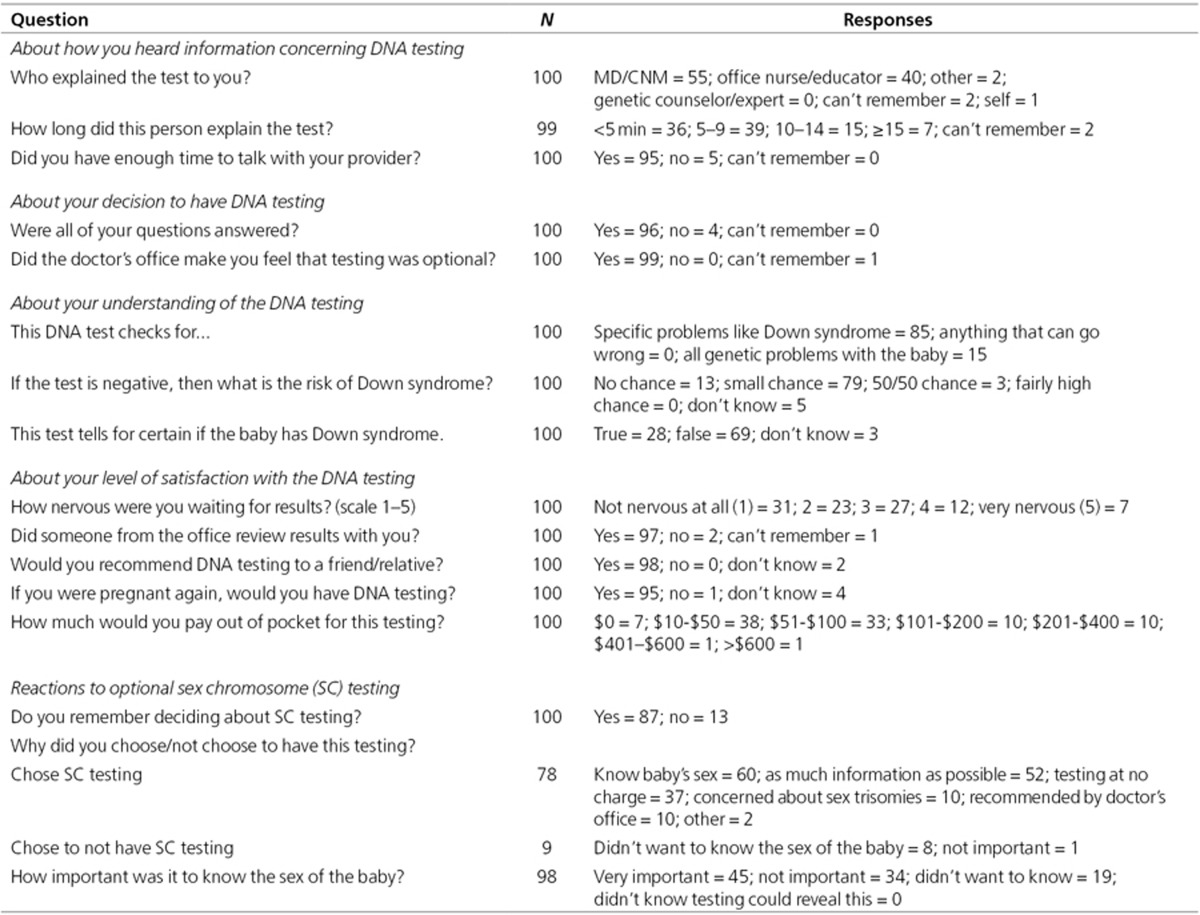
Summary of responses to selected questions from the patient survey
