# Supplementary material for: The clinical utility of DNA-based screening for fetal aneuploidy by primary obstetrical care providers in the general pregnancy population
Source: Genet Med. 2017 Jan 12;19(7):778–86. doi: 10.1038/gim.2016.194 (PMC5532959; doi:10.1038/gim.2016.194)
Supplement: Supplemental Materials [file gim2016194x1.doc]

**Supplemental Materials**

Supplemental Methods

Figure s1. The ACCE wheel (Analytic validity, Clinical validity, Clinical utility and Ethical legal and social implications)

Table s1. Standardized questions associated with the ACCE review structure

Figure s2. Sample DNA*First* requisition

Figure s3. Sample DNA*First* report

Figure s4. Patient education pamphlet

Figure s5. Maternal weight, gestational age and type of cfDNA test failure

Figure s6. Sex chromosome testing result

**Supplemental Methods**

**The DNA*First* Test:**

Two 10 mL plasma samples (Streck, Omaha, NE) and the test requisition were received at the WIH laboratory and shipped overnight to a commercial laboratory using a SNP genotyping methodology (Natera, Inc., San Carlos, CA).^1,2^ The accompanying serum sample was kept locally. DNA*First* reports were available within 24 hours after receipt of electronic results. Patient risks were capped at 9:1 (90%) to reinforce that cfDNA testing was a screening, not a diagnostic, test. Primary care providers were not trained to use on-line software^3^ to provide estimated PPVs. If cfDNA testing failed, options included submitting a second plasma sample, approving reflexive serum screening, or both. A cfDNA test was considered a failure, if it did not provide an actionable interpretation, even though the sample fulfilled inclusion criteria. Thus, a sample associated with known twin pregnancy, insufficient volume, or delayed transit would not be considered a test failure, but would be documented as failing inclusion criteria.

**Enrolling and Educating Primary Obstetrical Care Providers:**

Local program staff invited providers to participate, beginning with the larger group practices. Orientations occurred at provider staff meetings, sometimes accompanied by a light lunch or snack. Content discussed included: cfDNA testing methodology, no insurance or patient charge and potential responses to patient questions. Similarities to serum screening protocols were stressed (*e.g.,* local phlebotomy stations, similar requisitions and reports, and provider responsibility for patient consent for laboratory testing) and differences were highlighted (*e.g.,* low rate of screen positives, higher risks in screen positives, potential for cfDNA test failures and availability of sex trisomy/fetal sex testing). “Quick Reference” guides were distributed and a monthly newsletter encouraged and updated providers. Phlebotomists were also oriented and provided with customized drawing kits and instructions.

**Patient Materials***:*

The patient education brochure was brief, focused, and suitable for use by a generalist rather than genetic specialist. It addressed content recommendations by professional societies^4^ and was reviewed by focus groups of pregnant women. Reading level was 8^th^ to 9^th^ grade and the SAM (Suitability Assessment of Materials) evaluation grade that includes content, literacy demand, graphics and layout was 73%; classified as Superior.^5^ The final amended version (Figure s3) was IRB approved, translated into Spanish and distributed.

**Patient Survey***:*

Survey validation was accomplished by assessment and revision after content expert review, pilot testing of the questionnaire and subsequent review, and the inclusion of cross validating questions (same question asked in similar ways, or asked to both patients and providers). Inclusion criteria were: a signed ‘permission to contact’ on the requisition, 18 years of age or older, gestation between 10 and 20 weeks’ inclusive, and their provider offered DNA*First* for >8 weeks. Screen positive women were excluded due to their likelihood of receiving post-result genetic counseling that would interfere with the evaluation of information from their primary provider. Among all eligible candidates, one was randomly selected from each provider represented. Those with DNA test failures or those not choosing sex chromosome testing (relatively low percentages for both were expected) were over-selected. Among remaining candidates, random selection completed the list. An experienced genetic counselor (EMK) administered nearly all surveys. The exception was for those few women most comfortable with Spanish, where a fluent, hospital-based research professional was trained to use the interview script. Contact was first attempted during working hours. If unsuccessful after two or three calls during working hours, additional contacts were attempted in the evenings (6 - 8 pm) and on weekends (10 am to 8 pm). At least six attempts were made for each woman.

**Provider Survey***:*

At the project’s conclusion, a questionnaire was distributed to active providers focusing on their readiness to offer testing, patient education, perceived patient satisfaction and their patients’ concerns about the DNA*First* testing program. No incentives were provided for completion.

**References**

1. Nicolaides KH, Syngelaki A, Gil M, Atanasova V, Markova D. Validation of targeted sequencing of single-nucleotide polymorphisms for non-invasive prenatal detection of aneuploidy of chromosomes 13, 18, 21, X, and Y. *Prenat Diagn* 2013;33:575-579.

2. Pergament E, Cuckle H, Zimmermann B, Banjevic M, Sigurjonsson S, Ryan A, et al. Single-nucleotide polymorphism-based noninvasive prenatal screening in a high-risk and low-risk cohort. *Obstet Gynecol* 2014;124:210-218.

3. National Society of Genetic Counselors. NIPT/Cell Free DNA Screening Predicitve Value Calculator https://www.perinatalquality.org/Vendors/NSGC/NIPT/, August 24, 2016.

4. Kloza EM, Haddow PK, Halliday JV, O'Brien BM, Lambert-Messerlian GM, Palomaki GE. Evaluation of patient education materials: the example of circulating cell free DNA testing for aneuploidy. *J Genet Couns* 2015;24:259-266.

5. Doak CC, L.G. D, Root JH. Teaching Patients with Low Literacy Skills. Philadelphia, PA:JB Lippincott, 1996.

**Figure s1. The ACCE wheel.** The Analytic validity, Clinical validity, Clinical utility and Ethical legal and social implications (ACCE) are the four main components needed for a comprehensive public health assessment of screening tests. The process begins with a clear definition of the disorder of interest and the setting in which testing will be offered (the ‘hub’ of the wheel). Each component then works from the center of the wheel, with key elements of each component described. Systematic implementation of the ACCE model process is achieved by using a standardized set of 44 questions (contained in Table s1).

**Table s1. Standardized questions associated with the ACCE review structure**

Disorder/Setting

Disorder

1. What is the specific clinical disorder to be studied?
2. What are the clinical findings defining this disorder?

Setting

1. What is the clinical setting in which the test is to be performed?

Testing

1. What DNA test(s) are associated with this disorder?
2. Are preliminary screening questions employed?
3. Is it a stand-alone test or is it one of a series of tests?
4. If it is part of a series of tests, are all tests performed in all instances (parallel) or are only some tests performed on the basis of other results (series)?

Analytic Validity

1. Is the test qualitative or quantitative?

Sensitivity

1. How often is the test positive when a mutation is present (by person or by chromosome)?

Specificity

1. How often is the test negative when a mutation is not present?

Quality Control

1. In an internal QC program defined and externally monitored?
2. Have repeated measurements been made on specimens?
3. What is the within- and between-laboratory precision?
4. If appropriate, how is confirmatory testing performed to resolve false positive results in a timely manner?

Robustness

1. What range of patient specimens has been tested?
2. How often does the test fail to give a useable result?
3. How similar are results obtained in multiple laboratories using the same, or different, technology?

Clinical Validity

Sensitivity

1. How often is the test positive when the disorder is present?

Specificity

1. How often is the test negative when a disorder is not present?
2. Are there methods to resolve clinical false positive results in a timely manner?

Prevalence

1. What is the prevalence of the disorder in this setting?
2. Has the test been adequately validated on all populations to which it may be offered?

Positive/Negative Predictive Values

1. What are the positive and negative predictive values?

Penetrance

1. What are the genotype/phenotype relationships?
2. What are the genetic, environmental or other modifiers?

Clinical Utility

Natural History

1. What is the natural history of the disorder?

Intervention

1. What is the impact of a positive (or negative) test on patient care (including does the provision of the information in the absence of available treatment provide psychological benefit or lead to risk-reducing behavior?).
2. If applicable, are diagnostic tests available?
3. Is there an effective remedy or acceptable action?
4. Is there general access to that remedy or action?
5. Is the test being offered to a socially vulnerable population?

Quality Assurance

1. What quality assurance measures are in place?

Pilot Trials

1. What are the results of the pilot trials?

Health Risks

1. What health risks can be identified for follow-up testing and/or intervention?

Economic

1. What are the financial costs associated with testing?
2. What are the economic benefits associated with actions resulting from testing?

Facilities

1. What facilities/personnel are available or easily put in place?

Education

1. What educational materials have been developed and validated, and which of these are available?
2. Are there informed consent requirements?

Monitoring

1. What methods exist for long-term monitoring?
2. What guidelines have been developed for evaluation program performance?

Ethical, Legal and Social Implications (ELSI)

Impediments

1. What is known about stigmatization, discrimination, privacy/confidentiality and personal/family social issues?
2. Are there legal issues regarding consent, ownership of data and/or samples, patents, licensing, proprietary testing, obligation to disclose or reporting requirements?

Safeguards

1. What safeguards have been described and are these safeguards in place and effective?

**Figure s2. DNA*First* test requisition**


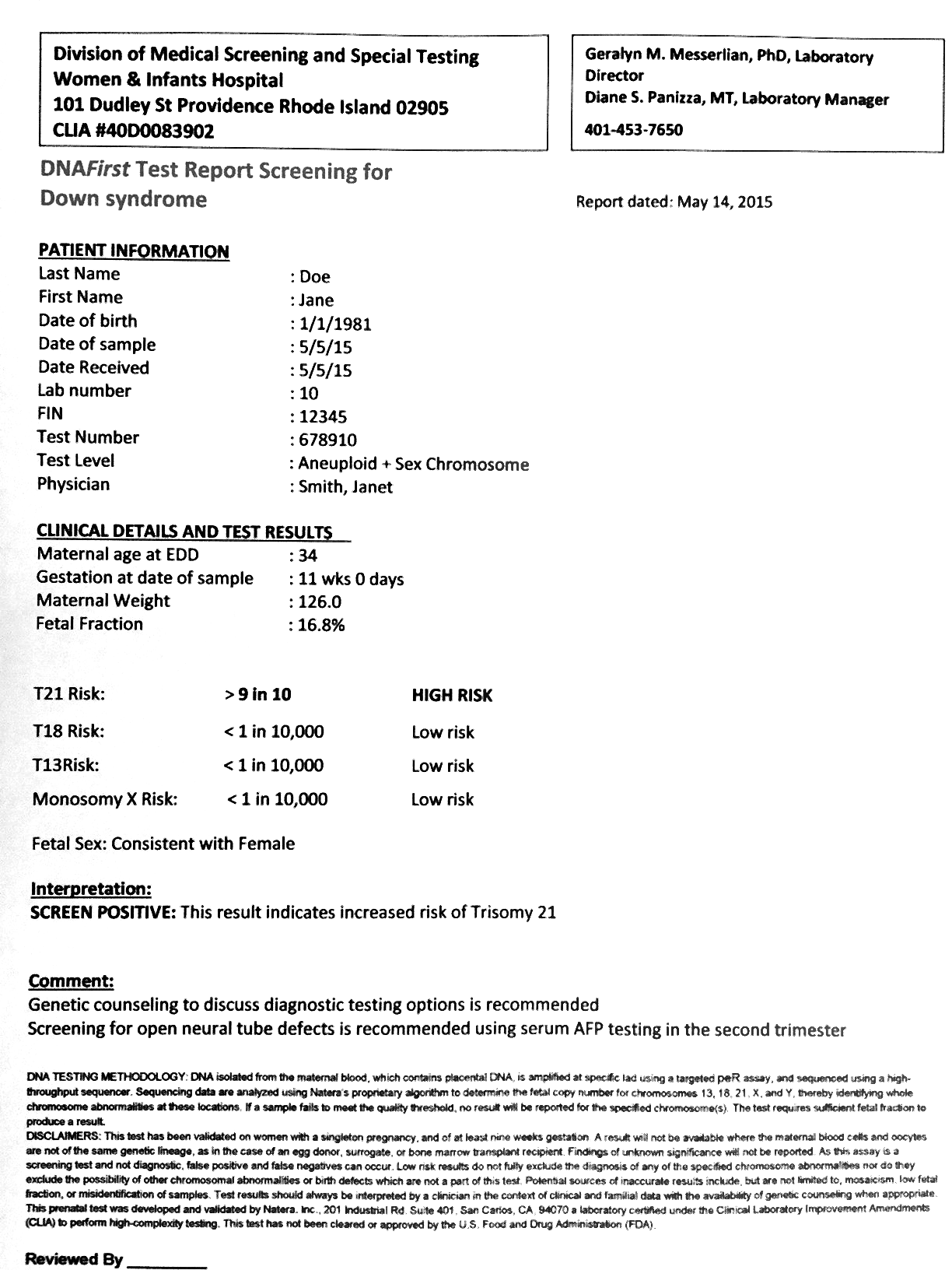


**Figure s3. Sample DNA*First* report.** A cfDNA screen positive report for trisomy 21.


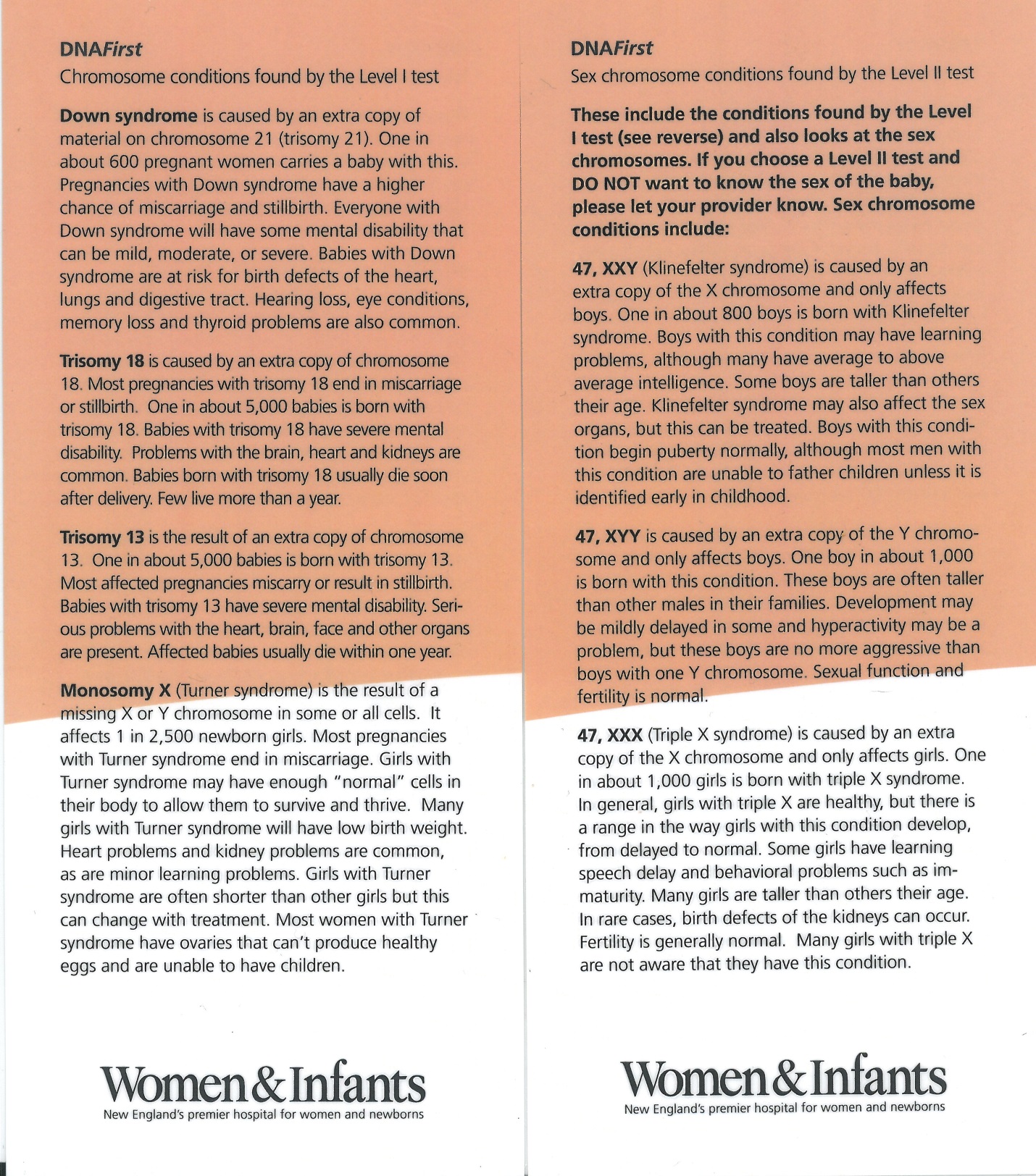


**Figure s4. Trifold patient education pamphlet (with insert)**

**Figure s4. Maternal weight versus gestational age and type of cfDNA test failure.** The gestational age is shown on the x-axis and the maternal weight on the y- axis. Missing weights are shown across the bottom of the y-axis. Successful initial tests are shown as small grey circles. Filled black circles are failures due to low fetal fraction and open circles are failures due to poor quality control metrics; other test failures are open squares. Among the 1,901 women below 80 Kg (dashed line), 14, 20 and 56 had failures due to low fetal fraction, poor quality metrics (*e.g.,* not enough DNA, too few SNP reads, too few informative SNPs) or for other reasons (*e.g.,* higher than expected levels of homozygosity, suspected fetal mosaicism, maternal chromosomal abnormalities or mosaicism, or suspected large deletion or duplication). Among the 612 women above that cut-off, failures occurred in 46, 24 and 18 women, respectively. Failures among women with missing weights were not included in this analysis**.**

**Figure s6. Flowchart showing the sex chromosome testing results, including the proportion of women opting for sex chromosome (and fetal sex) testing.** All 2,681 reported results were interpreted for monosomy X (Turner syndrome). Overall, 2,445 women opted for sex trisomy testing, two were screen positive and both received genetic counseling. Most failed test results were ‘complete’ failures already reported as part of trisomy 21, 18 and 13 screening performance.
